# Marizomib (Salinosporamide A) Promotes Apoptosis in A375 and G361 Melanoma Cancer Cells

**DOI:** 10.3390/md22070315

**Published:** 2024-07-15

**Authors:** Wiktoria Monika Piskorz, Rafał Krętowski, Marzanna Cechowska-Pasko

**Affiliations:** Department of Pharmaceutical Biochemistry, Medical University of Bialystok, Mickiewicza 2A, 15-222 Białystok, Poland

**Keywords:** proteasome inhibition, apoptosis, cancer treatment, salinosporamide A, NPI-0052

## Abstract

Malignant melanoma—a tumor originating from melanocytes—is characterized by dynamic growth and frequent metastases in the early stage of development. Current therapy methods are still insufficient, and there is a need to search for new ways of treating this malady. The induction of apoptosis—physiological cell death—by proteasome inhibitors is recognized as an effective method of non-invasive elimination of cancer cells. In our research, we wanted to check the potential of marizomib (MZB, salinosporamide A, NPI-0052)—an irreversible proteasome inhibitor derived from the marine actinomycete *Salinispora tropica*—to induce apoptosis in A375 and G361 malignant melanoma cells. We determined the cytotoxic activity of marizomib by performing an MTT test. Ethidium bromide and acridine orange staining demonstrated the disruption of membrane integrity in the examined cell lines. We confirmed the proapoptotic activity of marizomib by flow cytometry with the use of an FITC-Annexin V assay. A Western blot analysis presented an increase in the expression of proteins related to endoplasmic reticulum (ER) stress as well as markers of the apoptosis. The gathered findings suggest that marizomib induced the ER stress in the examined melanoma cancer cells and directed them towards the apoptosis pathway.

## 1. Introduction

Malignant melanoma is a skin cancer caused by malignancy of melanocytes. Characterized by dynamic growth and frequent metastases, it is considered the most lethal skin tumor. The number of new cases of this cancer increases every year, especially in Caucasians [1,2,3]. Treatment of malignant melanoma has improved significantly in recent years, especially with the introduction of therapies such as CTLA4, PD1 and BRAF inhibitors. However, in some cases, secondary resistance has developed. Additionally, the treatment of melanoma brain metastases still poses a challenge due to the blood–brain barrier [4,5]. Current treatment strategies are still insufficient. The search for new compounds effective against malignant melanoma is necessary for the development of new therapeutic strategies.

Cancer chemotherapy usually involves inhibiting cancer cell proliferation and inducing apoptosis—programmed cell death. In contrast to necrosis, apoptosis is a routine physiological process that occurs without causing an inflammation [6]. Its inhibition constitutes a basis for the development of many cancer diseases. 

According to reports, the initiation of apoptosis is possible, among other ways, by inhibiting the activity of the proteasome [7,8]. The proteasome is an enzyme complex responsible for regulating protein degradation. It consists of two external α subunits, responsible for proteolytic selectivity, and two internal β subunits, lysing ubiquitin-tagged proteins [9]. Proteasome activity influences the functioning of the entire cell, including the cell cycle, signal transduction, cell division and cellular homeostasis [10]. It also has a beneficial effect on the development of cancer. Uncontrolled cancer progression is associated with genetic mutations, abnormal RNA splicing and accelerated protein synthesis. Many of these proteins are misfolded and defective. Further growth of a cancer cell requires the removal of damaged proteins by the proteasome [11]. Proteasome inhibitors, by blocking proteolytically active subunits of the proteasome, increase endoplasmic reticulum (ER) stress and inactivate survival pathways [12]. 

Under normal conditions, protein kinase RNA-like endoplasmic reticulum kinase (PERK) is associated with immunoglobulin heavy chain-binding protein (BiP), also known as 78-kDa glucose-regulated protein (GRP78). Due to the ER stress, PERK dissociates from BiP/GRP78 proteins and undergoes autophosphorylation, which results in eukaryotic initiation factor 2 alpha (EIF2α) phosphorylation. The activating transcription factor 4 (ATF4) is activated and leads to C/EBP homologous protein (CHOP) activation [13]. Research indicates that CHOP can induce apoptosis in both intrinsic/mitochondrial and extrinsic/death receptor-mediated pathways. In the intrinsic pathway, CHOP influences the balance between proapoptotic and antiapoptotic proteins [14]. Antiapoptotic proteins, such as B-cell lymphoma 2 (BCL-2), B-cell lymphoma-extra large (BCL-XL) and myeloid cell leukemia sequence 1 (MCL-1), inhibit the release of cytochrome c, while proapoptotic proteins, like BCL-2-associated X protein (BAX), BH3-interacting domain death agonist (BID), BCL-2 interacting mediator of cell death (BIM) and phorbol-12-myristate-13-acetate-induced protein 1 (NOXA), promote the cytochrome c release [15]. The activity of CHOP upregulates the expression of BIM, BAX and BAK and downregulates the expression of MCL-1, BCL-2 and BCL-XL [14]. The predominance of proapoptotic proteins over antiapoptotic proteins leads to the release of cytochrome c from the mitochondria into the cytoplasm. The complex from apoptotic protease activating factor 1 (APAF1), procaspase-9 and released cytochrome c is formed. The cleavage of caspase-9 occurs, which contributes to the activation of effector caspases-3, -6 and -7 [15]. Effector caspases degrade enzymatic and structural proteins and lead to cell disintegration. Ligands for phagocytic cells are expressed and apoptotic bodies are formed and then digested by phagocytic cells [16].

In the extrinsic pathway, CHOP can bind to the death receptor pathway and enhance the expression of death receptor 4 (DR4) and 5 (DR5), which leads to caspase-8 activation [17]. BID is cleaved to t-BID and translocated to the mitochondria, where it promotes cytochrome c release and activation of effector caspases [14,15].

Marizomib (MZB) is a proteasome inhibitor derived from the marine actinomycete *Salinispora tropica*, able to permanently block three proteasome activities: caspase-like (C-L, β1), trypsin-like (T-L, β2), and chymotrypsin-like (CT-L, β5) [18,19]. Preclinical studies suggested greater effectiveness and safety of MZB compared to other proteasome inhibitors [20]. Its anticancer effect was observed, among others, in studies on breast cancer cells [21] and in phase I trials for the treatment of multiple myeloma [22]. Moreover, the ability of MZB to cross the blood–brain barrier has been proven, which expands the range of its therapeutic possibilities [20]. Considering the promising effects of MZB [23], we decided to check its activity against malignant melanoma. The aim of our research was to confirm whether MZB has cytotoxic and/or proapoptotic activity on G361 and A375 melanoma cancer cell lines. To evaluate it, we decided to perform the MTT test, the Fluorescein Isothiocyanate (FITC)-Annexin V and propidium iodide (PI) assay, staining with ethidium bromide and acridine orange, and an analysis of the expression of apoptosis-related and ER-stress-related proteins by Western blot.

## 2. Results

### 2.1. The Effect of Marizomib on Cell Viability

**Figure 1:**
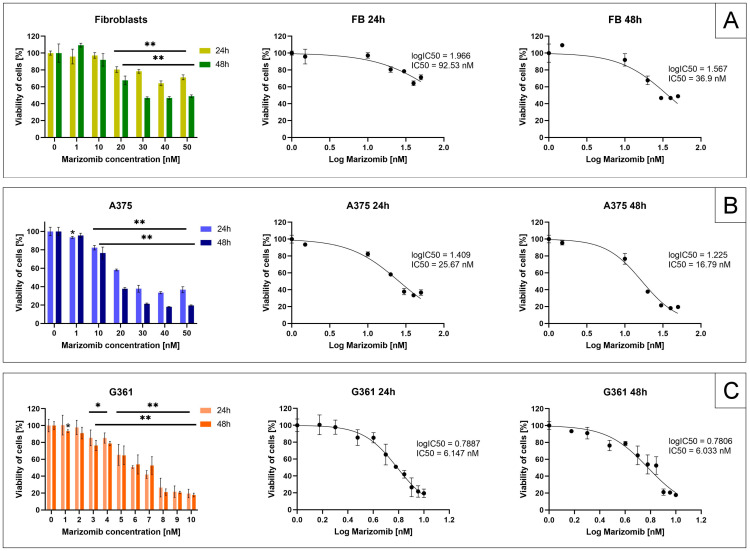
The effect of marizomib on the viability of fibroblasts (panel (**A**)) and A375 (panel (**B**)) and G361 (panel (**C**)) melanoma cells. Cells were incubated with MZB for 24 and 48 h. The A375 cells and fibroblasts (FB) were treated with MZB concentrations ranging from 1 to 50 nM and the G361 cells from 1 to 10 nM. The viability of the researched cells was calculated as a percentage of untreated cells (control). Mean values ± SD from three independent experiments performed in triplicate are presented. Significant alterations are marked with asterisks: * *p* < 0.05, ** *p* < 0.001.

The cytotoxic activity of MZB on G361 and A375 melanoma cells and human fibroblasts was evaluated by the MTT test (Figure 1). Cells were treated with MZB concentrations ranging from 1 nM to 50 nM in the melanoma A375 cell line and fibroblasts and from 1 nM to 10 nM in the melanoma G361 cell line and were incubated for 24 h and 48 h. Reduction in the viability of treated cells was visible in both cell lines. The obtained results were used to calculate the half-maximal inhibitory concentration (IC_50_) after 24 and 48 h of incubation with MZB. Fibroblasts turned out to be the most viable of the researched cells after contact with the inhibitor (panel A). The IC_50_ value for fibroblasts after 24 h of incubation was 92.53 nM and after 48 h was 36.9 nM. For the A375 line (panel B), the half-maximal inhibitory concentration after 24 h was 25.67 nM and after 48 h was 16.79 nM. The G361 line showed the lowest viability (panel C), with an IC_50_ value of 6.147 nM after 24 h and 6.033 nM after 48 h.

### 2.2. The Effect of Marizomib on Apoptosis

**Figure 2:**
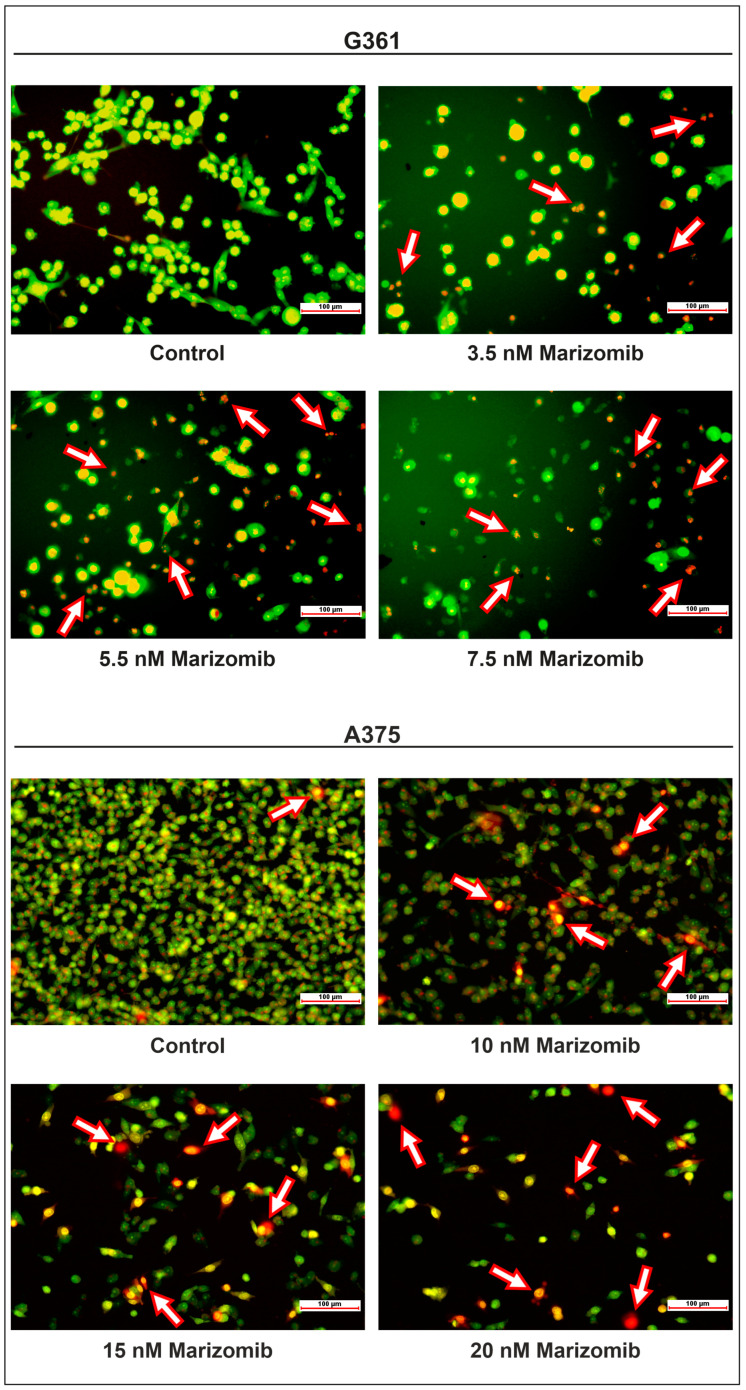
The effect of marizomib on the apoptosis of G361 and A375 melanoma cells. Cells were incubated with MZB for 48 h. The chosen MZB concentration ranged from 3.5 to 7.5 nM in the G361 line and from 10 to 20 nM in the A375 line. To identify living and apoptotic cells, the cells were stained with ethidium bromide and acridine orange and imaged using fluorescence microscopy (magnification 20×). Apoptotic cells are indicated with arrows.

Fluorescence microscopy and flow cytometry were used to investigate the proapoptotic effect of marizomib on melanoma G361 and A375 cell lines. The G361 cells were incubated with MZB concentrations ranging from 3.5 to 7.5 nM and the A375 cells from 10 to 20 nM for 48 h. Cells were then stained with ethidium bromide and acridine orange (Figure 2). Viable cells exhibited green nuclei without chromatin condensation. In apoptotic cells, nuclei were red and chromatin fragmentation was visible. Moreover, apoptotic bodies were also observed (apoptotic cells are marked with arrows).

**Figure 3:**
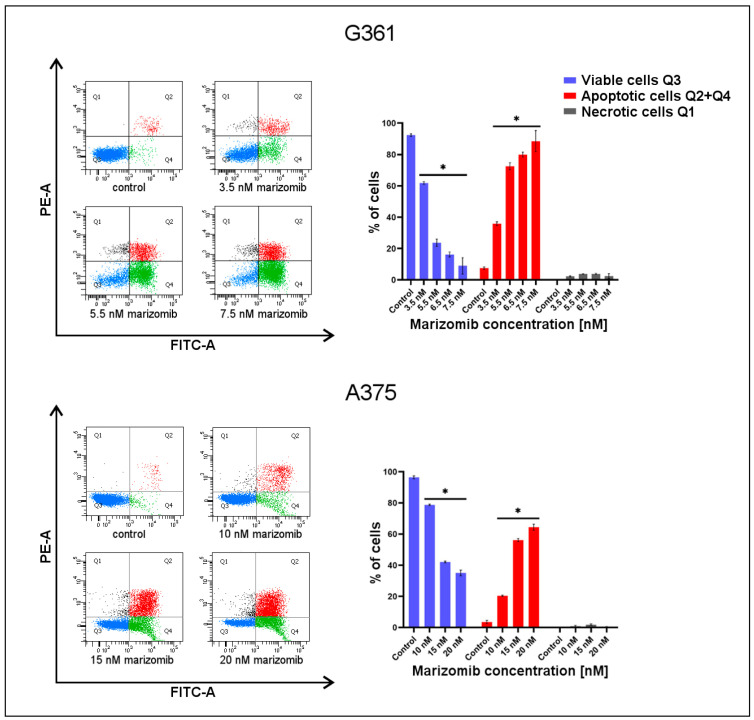
The effect of marizomib on the apoptosis of melanoma G361 and A375 cells. The cells were incubated with various concentrations of MZB for 48 h. The concentrations used ranged from 3.5 to 7.5 nM in the G361 line and from 10 to 20 nM in the A375 line. Subsequently, cells were analyzed by flow cytometry with the Annexin V-FITC and PI assay. Representative FACS images are presented on the left. Bar graphs show the percentage of apoptotic cells as a sum of the Q2 and Q4 quadrants and necrotic cells as the Q1 quadrant. Significant alterations in flow cytometry analysis are expressed relative to controls and are marked with asterisks (*). Results were gathered from three different experiments. Statistical significance is considered if * *p* < 0.001.

To further determine the apoptotic effect of MZB on the tested cells, we conducted a flow cytometry assay with FITC-Annexin V and PI (Figure 3). Viable cells bind neither to Annexin V nor to PI (quadrant Q3). Early apoptotic cells bind to Annexin V only (quadrant Q4), and late apoptotic cells bind to both Annexin V and PI (quadrant Q2). Necrotic cells are attached to PI only (quadrant Q1). Bar graphs represent the percentage of cells of each group depending on the MZB concentration. Early- (quadrant Q4) and late-phase (quadrant Q2) apoptotic cells were summed and presented in the graph as "Apoptotic cells Q2 + Q4". In both cell lines, there was a noticeable decrease in the percentage of viable cells (quadrant Q3) and a significant increase in apoptotic cells (quadrants Q2 + Q4). In the G361 line, the highest percentage of apoptotic cells (89% ± 6.8%) and the lowest percentage of viable cells (9% ± 5.2%) were observed at the 7.5 nM MZB concentration. In the A375 line, the largest increase in the percentage of apoptotic cells (64.4% ± 2.1%) with the greatest decrease in viable cells (35.1% ± 1.9%) occurred in at 20 nM of MZB.

### 2.3. The Effect of Marizomib on ER Stress

**Figure 4:**
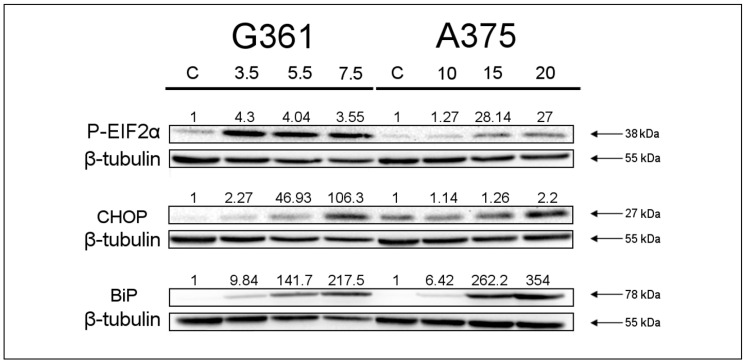
Western blot analysis of P-EIF2α, CHOP and BiP expression in melanoma cell lines treated with marizomib for 48 h. The G361 cells were incubated with concentrations from 3.5 to 7.5 nM and the A375 cells with concentrations from 10 to 20 nM. The concentrations used are presented at the top. The representative bands of P-EIF2α, CHOP, BiP and β-tubulin are illustrated. Samples containing 15 μg of protein were submitted to electrophoresis and immunoblotting. The densitometric analysis is presented as the relative fold change in comparison to untreated controls (C), where the expression level was set as 1. β-tubulin expression (55 kDa) was used as a loading control.

To investigate whether the changes occurring in MZB-treated cells were induced by ER stress, we performed a Western blot analysis to determine the expression of P-EIF2α, CHOP and BiP proteins (Figure 4). The results showed an increase in the expression of all three ER-stress-related proteins after 48 h of MZB treatment in both cell lines. The acquired data suggest that marizomib may affect ER homeostasis and induce ER stress in A375 and G361 cells in a concentration-dependent manner.

### 2.4. The Effect of Marizomib on the Expression of Apoptosis Markers

**Figure 5:**
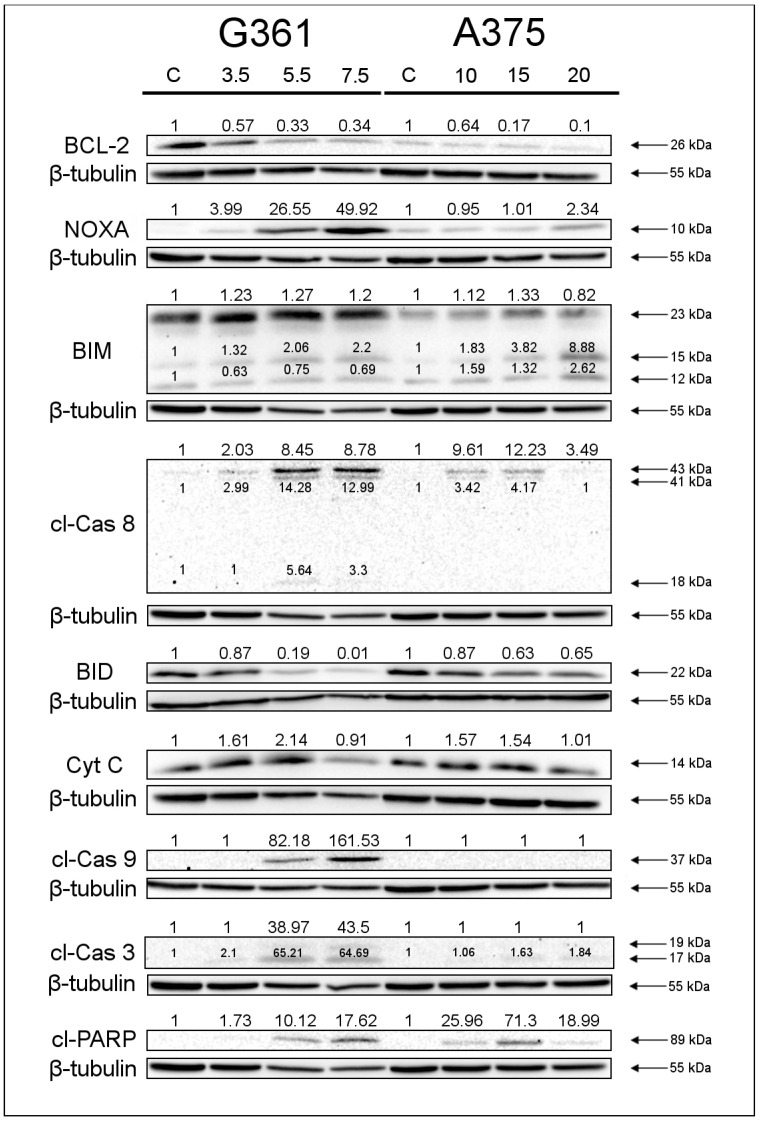
Western blot analysis of apoptosis marker levels in G361 and A375 melanoma cells after 48 h incubation with marizomib. The concentration used ranged from 3.5 to 5.5 nM in the G361 line and from 10 to 20 nM in the A375 line. Samples containing 15 μg of protein were submitted to electrophoresis and immunoblotting. The densitometric analysis is presented as the relative fold change in comparison to untreated controls (C), where the expression level was set as 1. β-tubulin expression (55 kDa) was used as a loading control.

To determine the effect of marizomib on the apoptosis of the researched malignant melanoma cell lines, an analysis of apoptosis marker levels was performed (Figure 5). The Western blot analysis showed a decrease in the expression of antiapoptotic protein BCL-2 and an increase in the proapoptotic protein NOXA in both cell lines. Moreover, a higher level of the proapoptotic protein BIM (isoforms BIM_EL_ and BIM_L_) was detected in both cell lines. An increase in the expression of the BIM_S_ isoform (12 kDa) was visible in the A375 line. The level of cleaved caspase-8 increased, while the expression of BID—which is cleaved by caspase-8 in the extrinsic apoptosis pathway—decreased. A higher level of cytochrome c was also visible. Cleaved caspase-9 was detected only in the G361 line, and its expression increased with MZB concentration. A higher level of cleaved caspase-3 was detected in both cell lines. One of the main cleavage targets of caspase-3 is poly (ADP-ribose) polymerase (PARP). An increased level of cleaved PARP was observed in both cell lines.

## 3. Discussion

Malignant melanoma is a high-grade tumor that originates from the pigment cells of the skin. It can metastasize both to nearby lymph nodes and to distant organs such as the lungs or brain. Due to the speed of metastasis formation, melanoma therapy requires rapid and effective action. Scientists are still looking for new, efficient methods of treating this cancer. Some of them are based on the induction of apoptosis—natural cell death. A promising group of chemotherapeutics are proteasome inhibitors, and among them marizomib, a natural proteasome inhibitor from the marine actinomycete *Salinispora tropica*, which has the ability to penetrate the blood–brain barrier and may prove useful in the treatment of malignant melanoma [24].

In our studies, we decided to determine the proapoptotic activity of MZB against malignant A375 and G361 melanoma cell lines and examine its mechanism of action. To evaluate it, MTT cytotoxicity tests, fluorescence microscopy, flow cytometry analyses and Western blot analyses were performed. 

Millward et al. [25] examined the ability of marizomib to inhibit the growth of lung tumors using the 3-(4,5-Dimethylthiazol-2-yl)-5-(3-carboxymethoxyphenyl)-2-(4-sulfophenyl)-2H-tetrazolium (MTS) assay. The IC_50_ values they determined varied depending on the cell line and ranged from 10 nM to 300 nM. Raninga et al. [21] concluded in their research that MZB reduced the proliferation of triple-negative breast cancer (TNBC) cells in a concentration-dependent manner, without much effect on non-TNBC and non-malignant mammary MCF10A and D492 epithelial cell lines. The gained IC_50_ values for MZB were less than 150 nM in TNBC lines and greater than 1 µM in non-TNBC lines. Yoo et al. [26] established the IC_50_ value of MZB in XO8 glioma-derived cancer stem cells, and it was 29 nM. To evaluate the IC_50_ value, we performed the MTT test on malignant melanoma A375 and G361 cell lines and normal fibroblasts. The test showed that melanoma cells are more sensitive to the action of marizomib than fibroblasts. In fibroblasts, the IC_50_ values were higher than in melanoma cell lines after both 24 h (92.53 nM) and 48 h (36.9 nM) of incubation with MZB. In the A375 melanoma cell line, the IC_50_ value was 25.67 nM after 24 h and 16.79 nM after 48 h. Among the cell lines we tested, the G361 line turned out to be the most susceptible to the cytotoxic effect of MZB, with an IC_50_ value of 6.147 nM after 24 h and 6.033 nM after 48 h. Different sensitivities of G361 and A375 cells to various compounds was also observed by Tuzimski et al. [27]. In this study, as in the case of MZB, the G361 line cells showed a lower IC_50_ value than A375 cells after contact with the *Sanguinaria canadensis* extracts berberine and etoposide. However, the opposite effect, where A375 cells had a lower IC_50_ value than G361 cells, was observed after the use of chelerythrine, sanguinarine or cisplatin. Based on these observations, it can be assumed that the differences in the response of the tested melanoma cells to the compound used are related to the factor and not to the overall greater sensitivity of G361 cells. Moreover, noting that in the G361 line, the IC_50_ values for MZB after 24 and 48 h are similar, it can be concluded that in the case of this cell line, the dose of MZB used had a greater impact on the cell response than the incubation time.

Numerous prospective medicinal substances have been found among aquatic species. Many of them may be effective in the fight against malignant melanoma, while being less harmful to normal cells [28]. In the case of the A375 line, the effect of marizomib can be compared to penicitrinine A—an alkaloid isolated from the marine fungus *Penicilium citrinum*. The IC_50_ values for the A375 line were equal to 30.88 μM after 24 h and 12.78 μM after 48 h incubation with penicitrinine A [29]. Researchers also presented that penicitrinine A induced apoptosis and suppressed the metastatic activity of A375 cells. Among the compounds of marine origin, active against A375 cells, there are also pyrenosetins A and B, with IC_50_ values of 2.8 and 6.3 μM, respectively [30]. Among the indicated products of marine origin, active against A375 cells, marizomib has the lowest IC_50_ value (expressed in nanomoles). However, the effectiveness of the tested compound in cancer therapy is determined by the appropriate balance between anticancer activity and toxicity towards healthy cells. Marizomib appears to be well tolerated by normal cells, as the level of its toxicity is higher in melanoma cells than in fibroblasts. However, further clinical trials are needed to clearly assess the safety of this inhibitor. 

The next step was to check whether the toxicity of marizomib towards melanoma cells was related to the induction of apoptosis. To evaluate it, we first used ethidium bromide and acridine orange. Photos taken using a fluorescence microscope in MZB-treated samples showed cells with signs of apoptosis: chromatin condensation, red nuclei and the formation of apoptotic bodies (Figure 2). To confirm the apoptosis, Annexin V-FITC and PI staining and flow cytometry analysis were used. The obtained dot plots (Figure 3) show a clear difference in the distribution of untreated and MZB-treated cells. As the concentration of MZB increased, the number of apoptotic cells increased and the number of viable cells decreased in both cell lines. The results confirm the proapoptotic activity of MZB on the tested cell lines. Di et al. [20] demonstrated that 60 nM of marizomib induced apoptosis and caspase-3 activation in glioma cells. Raninga et al. [21] performed in vivo tests and analyzed the percentage of apoptotic cells by ApopTag staining. They compared the 4T1.2 tumors treated with vehicle and MZB for two weeks. They observed an increased percentage of ApopTag-positive cells in MZB-treated tumor tissues, which also suggests that MZB induces apoptosis in vivo.

We checked the effect of MZB on ER stress by examining the levels of P-EIF, CHOP and BiP—ER-stress-related proteins. We were able to establish that MZB induces ER stress, leading to BiP, P-EIF and CHOP upregulation. Similar results were gained by Yoo et al. [26], who observed the upregulation of CHOP and BiP in MZB-treated XO8 glioma-derived cancer stem cells. 

To assess the pathway of apoptosis induced in treated cells, we performed Western blot analyses. The results showed that the effect of MZB resulted in a decrease in the level of the antiapoptotic protein BCL-2 and an increase in the proapoptotic proteins BIM and NOXA. Similar results were observed by Raninga et al. [21] in their studies on triple-negative breast cancer cells treated with marizomib. The researchers demonstrated a decrease in BCL-2 and MCL-1 levels and an increase in BIM proteins in treated cells. Moreover, they observed an increase in caspase-3 activity and enhanced cleavage of PARP. Our Western blot analysis also shows an upregulated expression of cleaved caspase-3 and cleaved PARP. Effector caspase-3 activity explains the proapoptotic changes observed in fluorescence microscopy and flow cytometry analyses. However, caspase-3 can be activated through various pathways, particularly the caspase-8 pathway and caspase-9 pathway. In the intrinsic pathway, there is an increased release of cytochrome c, which combines with caspase-9 and activates effector caspases (including caspase-3). In the extrinsic pathway, caspase-8 is activated, which cleaves BID to t-BID and promotes the release of cytochrome c in mitochondria. In our analyses, an increase in the level of cytochrome c was observed in both cell lines. Active caspase-9 was detected in the G361 line only. An increase in active caspase-8 and a simultaneous decrease in BID were noted in both tested cell lines. Manton et al. [31] proved that MZB induced caspase-8 and caspase-9 activation in glioblastoma. Miller et al. [32] observed the caspase-8 activation in their research on activity of MZB on leukemia systems. Moreover, they suggest that MZB-dependent apoptosis relies more heavily on a caspase-8 pathway.

Our results suggest that MZB has a proapoptotic effect on malignant melanoma G361 and A375 cell lines. MZB leads to ER stress and increased CHOP levels. As a result, in both cell lines, the extrinsic pathway of apoptosis is activated, which, through activation of caspase-8 and cleavage of BID, increases cytochrome c release and leads to caspase-3 activation. Moreover, our research shows that in the G361 line, apoptosis also occurs via the intrinsic pathway, where the release of cytochrome c leads to the activation of caspase-9, which activates caspase-3. The combination of bortezomib and IFN-alpha performed by Lesinski et al. [33] on melanoma cells suggests a similar mechanism of action. Researchers observed apoptosis associated with the activation of caspase-3, caspase-7, caspase-8 and caspase-9, as well as cleavage of BID and PARP, which is similar to our results of MZB activity against melanoma cells. Moreover, research conducted by Niewerth et al. [34] indicated the activity of MZB in the case of resistance to bortezomib, which significantly expands the therapeutic possibilities of MZB treatment. Ahn et al. [35] presented that marizomib also enhances the effect of other anticancer compounds. In their study, they determined that MZB upregulated TNF-induced apoptosis from 20% to 68% and potentiated the apoptotic activity of bortezomib and thalidomide. In addition, Boccellato et al. [36] proved that MZB sensitizes primary glioma cells to apoptosis induced by a latest-generation TRAIL receptor agonist. Our research shows that marizomib may be a promising therapeutic agent for the treatment of malignant melanoma. The collected literature and our results indicate that MZB may be effective as an anticancer ingredient both alone and in combination with other drugs.

## 4. Materials and Methods

### 4.1. Reagents

McCoy’s 5A Medium (ATCC 30-2007) was provided by the American Type Culture Collection (ATCC) (Manassas, VA, USA). Dulbecco’s modified Eagle’s medium (DMEM), containing glucose (4.5 mg/mL) with GlutaMax^TM^, fetal bovine serum Gold (FBS Gold), phosphate buffer saline (PBS), trypsin-EDTA, streptomycin and penicillin, was obtained from Gibco (San Diego, CA, USA). Marizomib was provided by MedChemExpress (New York city, NY, USA). The RIPA buffer and the Protease/Phosphatase Inhibitor Cocktail were products of Cell Signalling Technology (Boston, MA, USA). Both the Pierce^TM^ ECL Western Blotting Substrate and BCA Protein Assay Kit were ordered from Thermo Scientific (Rockford, IL, USA). Antibodies obtained from Cell Signalling Technology (Boston, MA, USA) were as follows: Phospho-EIF2α (Ser51) (D9G8) XPTM rabbit mAb, CHOP (D46F1) rabbit mAb, BiP (C50B12) rabbit mAb, BCL-2 (D17C4) rabbit mAb, NOXA (D8L7U) rabbit mAb, BIM (C34C5) rabbit mAb, Cleaved Caspase-8 (Asp374) (E6H8S) rabbit mAb, BID Antibody (Human Specific), Cytochrome c (D18C7) rabbit mAb, Cleaved Caspase-9 (Asp330) (E5Z7N) rabbit mAb, Cleaved Caspase-3 (Asp175) antibody, Cleaved PARP (Asp214) (D64E10) XP rabbit mAb and β-tubulin rabbit antibody. Immuno-Blot PVDF Membranes for Protein Blotting were products of Bio-Rad (Hercules, CA, USA).

### 4.2. Cell Culture and Exposure to Marizomib

The malignant melanoma cell lines G361 (ATCC CRL-1424; mutant genes: BRAF, CDKN2A, STK11) and A375 (ATCC CRL-1619, mutant genes: BRAF, CDKN2A) as well as human skin fibroblasts (CRL 1474) were obtained from the American Type Culture Collection (ATCC). The G361 cells were cultured in McCoy’s 5A Medium, while the A375 cells and fibroblasts were cultured in DMEM, both supplemented with penicillin (100 U/mL), streptomycin (100 µg/mL) and 10% heat-inactivated fetal bovine serum Gold (FBS Gold), in accordance with the manufacturer’s recommendations. Cells were cultured at 37 °C in an atmosphere enriched with 5% CO_2_, provided by a Galaxy S+ incubator (RS Biotech, Irvine, UK). To wash cells, DPBS (pH 7.4) was used. Confluent cells were detached with the use of 0.05% trypsin in 0.02% EDTA and counted in a Scepter Cell Counter (Millipore, MA, USA). The appropriate number of cells were seeded onto culture plates. After 24 h, culture media were replaced with fresh media containing marizomib. Cells were incubated for 48 h at 37 °C in an atmosphere enriched with 5% CO_2_. The number of passages performed did not exceeded 10.

### 4.3. Marizomib Concentrations

The A375 and G361 cell lines and fibroblasts were initially incubated in a wide range of marizomib concentrations (unpublished results). Our observations showed that from a concentration of 30 nM MZB, the average cell viability of the A375 cells was low and remained on a similar level, despite the increase in this inhibitor concentration. A similar effect was observed in the G361 line from the concentration of 20 nM MZB. Such low viability makes it difficult to conduct cell culture research. For this reason, we narrowed the concentrations in the A375 cell line to 1, 10, 20, 30, 40 and 50 nM. We used the same concentration range for fibroblasts. In the G361 cell line, the tested concentrations were narrowed down to 1–10 nM. In our research, we focused on examining the mechanism of apoptosis at the lowest possible effective concentrations of marizomib, which is of great importance in the eventual achievement of this inhibitor concentration in therapy in vivo. Therefore, the concentrations of MZB used in further analyses vary between cell lines and are close to the calculated IC_50_ value.

### 4.4. Cell Viability

In order to analyze the effect that marizomib has on cell viability, 3-(4,5-dimethylthiazol-2-yl)-2,5-diphenyltetrazolium bromide (MTT) was used according to the method of Carmichael [37]. The A375 and G361 cells and fibroblasts were seeded in 24-well plates (0.5 × 10^5^ cells per well). Subsequently, confluent cells were cultured with wide range of marizomib concentrations (1–10 nM in the G361 line and 1–50 nM in the A375 line and fibroblasts) for 24 and 48 h. Afterwards, the cells were washed with PBS (three times) and incubated with 1 mL of the MTT solution (0.25mg/mL in PBS) for 1 h (37 °C, 5% CO_2_). The medium was removed, and 0.5 mL of 0.1 mol/L HCl was added. The absorbance was measured on a microplate reader (Tecan, Männedorf, Switzerland) at a wavelength of 570 nm. The viability of A375 and G361 cells and fibroblasts was calculated as a percentage of control, untreated cells. All the experiments were performed in triplicates in three different cultures.

### 4.5. Fluorescent Microscopy

In order to analyze the nuclear morphology of apoptotic cells, melanoma cancer cells (lines A375 and G361) were stained with ethidium bromide and acridine orange. Cells were incubated with MZB for 48 h. The concentrations used ranged from 3.5 to 7.5 nM in the G361 line and from 10 to 20 nM in the A375 line. Then, cells were stained with the dye mixture, containing 15 μM acridine orange and 15 μM ethidium bromide in 1 mL DPBS (pH 7.4), for 10 min in the dark at room temperature. Subsequently, the staining solution was replaced with DPBS, and cells were washed twice. Ethidium bromide stained only cells that had lost their membrane integrity, while acridine orange stained both live and dead cells. The samples were analyzed with fluorescence microscopy (Olympus CXK41, U-RLFT50, Hamburg, Germany) according to the following criteria: living cells—normal green nucleus; early apoptotic cells—bright green nucleus with condensed or fragmented chromatin; and late apoptotic cells—orange-stained nuclei with chromatin condensation or fragmentation. Cells were analyzed at 20× magnification and photographed. Results were gathered from three different experiments.

### 4.6. Detection of Apoptosis and Necrosis

In order to evaluate the level of apoptosis and necrosis in A375 and G361 cells treated with MZB, the flow cytometry assay on an FACSCanto II cytometer (BD, San Diego, CA, USA) was used. The cells (2.0 × 10^5^) were seeded in six-well plates in 2 mL of culture media. After 24 h, the media were replaced with MZB concentrations ranging from 3.5 to 7.5 nM in the G361 line and from 10 to 20 nM in the A375 line and incubated for 48 h (37 °C, 5% CO_2_). Subsequently, cells were detached and centrifuged (1000 rpm for 5 min at room temperature) in order to remove the culture media. Samples were then washed with DPBS (pH 7.4) and resuspended in a binding buffer. The cells were then stained with FITC Annexin V and PI (FITC Annexin V apoptosis detection Kit I, BD PharmingenTM, San Diego, CA, USA) in the dark for 15 min at room temperature. Data were analyzed using FACSDiva software (BD PharmingenTM, San Diego, CA, USA). Results were gathered from three different experiments.

### 4.7. Total Protein Concentration in the Cells

The protein concentration in cell lysates was measured with a BCA Protein Assay Kit (Rockford, IL, USA) [38]. We used bovine serum albumin as a standard. The concentrations of the albumin standard were 25, 50, 100, 150, 200, 300 and 400 μg/mL.

### 4.8. Western Blot Analysis

The A375 and G361 cells were washed with cold PBS (pH 7.4) and solubilized in 300 μL of RIPA buffer with a protease-/phosphatase-inhibitor cocktail. Lysates were then centrifuged for 15 min (10,000× *g*, 4 °C) and analyzed for protein content. A 12% polyacrylamide gel was prepared. The samples containing 15 μg of protein were subjected to SDS-PAGE, according to Laemmli [39]. A constant current of 25 mA was used.

Subsequently, proteins were transferred to PVDF membranes and placed in blocking buffer (5% non-fat dry milk in Tris-buffered saline (TBS) containing 0.05% Tween 20 (TBS-T)) for 1 h. The membranes were then washed with TBS-T three times. Appropriate antibodies were dissolved in 5% non-fat dry milk in TBS (concentration 1:1000). Membranes were incubated with antibodies overnight at 4 °C. Next, membranes were rinsed with TBS-T. The HRP conjugated with anti-human secondary antibody against rabbit IgG at a 1:1000 dilution in TBS was added. After 2 h, the membranes were washed again with TBS-T and exposed to an electrochemiluminescence (ECL) reagent.

### 4.9. Chemiluminescence Detection

The membranes prepared for reading were treated with a luminol-based enhanced chemiluminescence substrate for peroxidase. The signal was recorded with GeneGnome (Syngene, Frederick, MD, USA) and transmitted to GeneTools software (Syngene, Frederick, MD, USA). Actual photos of the membranes are included in the Supplementary Materials.

### 4.10. Statistical Analysis

A statistical analysis was performed with the use of GraphPad Prism software version 9 (GraphPad Software, San Diego, CA, USA). Data were statistically analyzed by ANOVA followed by Tukey’s/Dunnett’s post hoc *t*-test analysis. Data were demonstrated as means ± standard deviations (SD). The significant differences of means were determined at the level of * *p* < 0.05.

## Data Availability

Data are contained within the article or Supplementary Material.

## Supplementary Materials

The following supporting information can be downloaded at https://www.mdpi.com/article/10.3390/md22070315/s1, Figure S1: Actual photos of Western blot membranes to Figure 4; Figure S2: Actual photos of Western blot membranes to Figure 5.

## References

[1] Dzwierzynski W.W. (2021). Melanoma Risk Factors and Prevention. Clin. Plast. Surg..

[2] Ahmed B., Qadir M.I., Ghafoor S. (2020). Malignant Melanoma: Skin Cancer-Diagnosis, Prevention, and Treatment. Crit. Rev. Eukaryot. Gene Expr..

[3] Santourlidis S., Schulz W.A., Araúzo-Bravo M.J., Gerovska D., Ott P., Bendhack M.L., Hassan M., Erichsen L. (2022). Epigenetics in the Diagnosis and Therapy of Malignant Melanoma. Int. J. Mol. Sci..

[4] Davis L.E., Shalin S.C., Tackett A.J. (2019). Current state of melanoma diagnosis and treatment. Cancer Biol. Ther..

[5] Jenkins R.W., Fisher D.E. (2021). Treatment of Advanced Melanoma in 2020 and Beyond. J. Investig. Dermatol..

[6] Xu X., Lai Y., Hua Z.C. (2019). Apoptosis and apoptotic body: Disease message and therapeutic target potentials. Biosci. Rep..

[7] Li J., Zhang X., Shen J., Guo J., Wang X., Liu J. (2019). Bortezomib promotes apoptosis of multiple myeloma cells by regulating HSP27. Mol. Med. Rep..

[8] Guo N., Peng Z. (2013). MG132, a proteasome inhibitor, induces apoptosis in tumor cells. Asia Pac. J. Clin. Oncol..

[9] Bańkowski E. (2017). Biochemistry Workbook for Students of the Faculty of Medicine and the Faculty of Health Sciences.

[10] Bard J.A.M., Goodall E.A., Greene E.R., Jonsson E., Dong K.C., Martin A. (2018). Structure and Function of the 26S Proteasome. Annu. Rev. Biochem..

[11] Chen Y., Zhang Y., Guo X. (2017). Proteasome dysregulation in human cancer: Implications for clinical therapies. Cancer Metastasis Rev..

[12] Nunes A.T., Annunziata C.M. (2017). Proteasome inhibitors: Structure and function. Semin. Oncol..

[13] Rozpedek W., Pytel D., Mucha B., Leszczynska H., Diehl J.A., Majsterek I. (2016). The Role of the PERK/eIF2α/ATF4/CHOP Signaling Pathway in Tumor Progression During Endoplasmic Reticulum Stress. Curr. Mol. Med..

[14] Hu H., Tian M., Ding C., Yu S. (2019). The C/EBP Homologous Protein (CHOP) Transcription Factor Functions in Endoplasmic Reticulum Stress-Induced Apoptosis and Microbial Infection. Front. Immunol..

[15] Kashyap D., Garg V.K., Goel N. (2021). Intrinsic and extrinsic pathways of apoptosis: Role in cancer development and prognosis. Adv. Protein Chem. Struct. Biol..

[16] D’Arcy M.S. (2019). Cell death: A review of the major forms of apoptosis, necrosis and autophagy. Cell Biol. Int..

[17] Lu M., Lawrence D.A., Marsters S., Acosta-Alvear D., Kimmig P., Mendez A.S., Paton A.W., Paton J.C., Walter P., Ashkenazi A. (2014). Opposing unfolded-protein-response signals converge on death receptor 5 to control apoptosis. Science.

[18] Macherla V.R., Mitchell S.S., Manam R.R., Reed K.A., Chao T.H., Nicholson B., Deyanat-Yazdi G., Mai B., Jensen P.R., Fenical W.F. (2005). Structure-activity relationship studies of salinosporamide A (NPI-0052), a novel marine derived proteasome inhibitor. J. Med. Chem..

[19] Potts B.C., Albitar M.X., Anderson K.C., Baritaki S., Berkers C., Bonavida B., Chandra J., Chauhan D., Cusack J.C., Fenical W. (2011). Marizomib, a proteasome inhibitor for all seasons: Preclinical profile and a framework for clinical trials. Curr. Cancer Drug Targets.

[20] Di K., Lloyd G.K., Abraham V., MacLaren A., Burrows F.J., Desjardins A., Trikha M., Bota D.A. (2016). Marizomib activity as a single agent in malignant gliomas: Ability to cross the blood-brain barrier. Neuro Oncol..

[21] Raninga P.V., Lee A., Sinha D., Dong L.F., Datta K.K., Lu X., Kalita-de Croft P., Dutt M., Hill M., Pouliot N. (2020). Marizomib suppresses triple-negative breast cancer via proteasome and oxidative phosphorylation inhibition. Theranostics.

[22] Ma L., Diao A. (2015). Marizomib, a potent second generation proteasome inhibitor from natural origin. Anticancer Agents Med. Chem..

[23] Pereira R.B., Evdokimov N.M., Lefranc F., Valentão P., Kornienko A., Pereira D.M., Andrade P.B., Gomes N.G.M. (2019). Marine-Derived Anticancer Agents: Clinical Benefits, Innovative Mechanisms, and New Targets. Mar. Drugs.

[24] Gozdz A. (2023). Proteasome Inhibitors against Glioblastoma-Overview of Molecular Mechanisms of Cytotoxicity, Progress in Clinical Trials, and Perspective for Use in Personalized Medicine. Curr. Oncol..

[25] Millward M., Price T., Townsend A., Sweeney C., Spencer A., Sukumaran S., Longenecker A., Lee L., Lay A., Sharma G. (2012). Phase 1 clinical trial of the novel proteasome inhibitor marizomib with the histone deacetylase inhibitor vorinostat in patients with melanoma, pancreatic and lung cancer based on in vitro assessments of the combination. Investig. New Drugs.

[26] Yoo Y.D., Lee D.H., Cha-Molstad H., Kim H., Mun S.R., Ji C., Park S.H., Sung K.S., Choi S.A., Hwang J. (2017). Glioma-derived cancer stem cells are hypersensitive to proteasomal inhibition. EMBO Rep..

[27] Tuzimski T., Petruczynik A., Plech T., Kaproń B., Makuch-Kocka A., Szultka-Młyńska M., Misiurek J., Buszewski B. (2021). Determination of Cytotoxic Activity of Sanguinaria canadensis Extracts against Human Melanoma Cells and Comparison of Their Cytotoxicity with Cytotoxicity of Some Anticancer Drugs. Molecules.

[28] Ahmed S., Alam W., Alsharif K.F., Aschner M., Alzahrani F.M., Saso L., Khan H. (2023). Therapeutic potential of marine peptides in malignant melanoma. Environ. Res..

[29] Liu Q.Y., Zhou T., Zhao Y.Y., Chen L., Gong M.W., Xia Q.W., Ying M.G., Zheng Q.H., Zhang Q.Q. (2015). Antitumor Effects and Related Mechanisms of Penicitrinine A, a Novel Alkaloid with a Unique Spiro Skeleton from the Marine Fungus *Penicillium citrinum*. Mar. Drugs..

[30] Fan B., Dewapriya P., Li F., Blümel M., Tasdemir D. (2020). Pyrenosetins A-C, New Decalinoylspirotetramic Acid Derivatives Isolated by Bioactivity-Based Molecular Networking from the Seaweed-Derived Fungus *Pyrenochaetopsis* sp. FVE-001. Mar. Drugs.

[31] Manton C.A., Johnson B., Singh M., Bailey C.P., Bouchier-Hayes L., Chandra J. (2016). Induction of cell death by the novel proteasome inhibitor marizomib in glioblastoma in vitro and in vivo. Sci. Rep..

[32] Miller C.P., Ban K., Dujka M.E., McConkey D.J., Munsell M., Palladino M., Chandra J. (2007). NPI-0052, a novel proteasome inhibitor, induces caspase-8 and ROS-dependent apoptosis alone and in combination with HDAC inhibitors in leukemia cells. Blood.

[33] Lesinski G.B., Raig E.T., Guenterberg K., Brown L., Go M.R., Shah N.N., Lewis A., Quimper M., Hade E., Young G. (2008). IFN-alpha and bortezomib overcome Bcl-2 and Mcl-1 overexpression in melanoma cells by stimulating the extrinsic pathway of apoptosis. Cancer Res..

[34] Niewerth D., Jansen G., Riethoff L.F., van Meerloo J., Kale A.J., Moore B.S., Assaraf Y.G., Anderl J.L., Zweegman S., Kaspers G.J. (2014). Antileukemic activity and mechanism of drug resistance to the marine *Salinispora tropica* proteasome inhibitor salinosporamide A (Marizomib). Mol. Pharmacol..

[35] Ahn K.S., Sethi G., Chao T.H., Neuteboom S.T., Chaturvedi M.M., Palladino M.A., Younes A., Aggarwal B.B. (2007). Salinosporamide A (NPI-0052) potentiates apoptosis, suppresses osteoclastogenesis, and inhibits invasion through down-modulation of NF-kappaB regulated gene products. Blood.

[36] Boccellato C., Kolbe E., Peters N., Juric V., Fullstone G., Verreault M., Idbaih A., Lamfers ML M., Murphy B.M., Rehm M. (2021). Marizomib sensitizes primary glioma cells to apoptosis induced by a latest-generation TRAIL receptor agonist. Cell Death Dis..

[37] Carmichael J., DeGraff W.G., Gazdar A.F., Minna J.D., Mitchell J.B. (1987). Evaluation of a tetrazolium-based semiautomated colorimetric assay: Assessment of chemosensitivity testing. Cancer Res..

[38] Smith P.K., Krohn R.I., Hermanson G.T., Mallia A.K., Gartner F.H., Provenzano M.D., Fujimoto E.K., Goeke N.M., Olson B.J., Klenk D.C. (1985). Measurement of protein using bicinchoninic acid. Anal. Biochem..

[39] Laemmli U.K. (1970). Cleavage of structural proteins during the assembly of the head of bacteriophage T4. Nature.

