# Supplementary material for: Marizomib (Salinosporamide A) Promotes Apoptosis in A375 and G361 Melanoma Cancer Cells
Source: Mar Drugs. 2024 Jul 15;22(7):315. doi: 10.3390/md22070315 (PMC11278368; doi:10.3390/md22070315)
Supplement: Supplementary file 1 [file marinedrugs-22-00315-s001.zip › marinedrugs-3093564-supplementary.pdf]

Supplement

Western blot marker used:

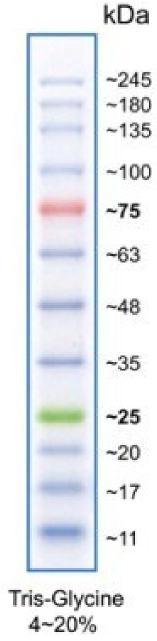

P-EIF2α (line G361/A375)

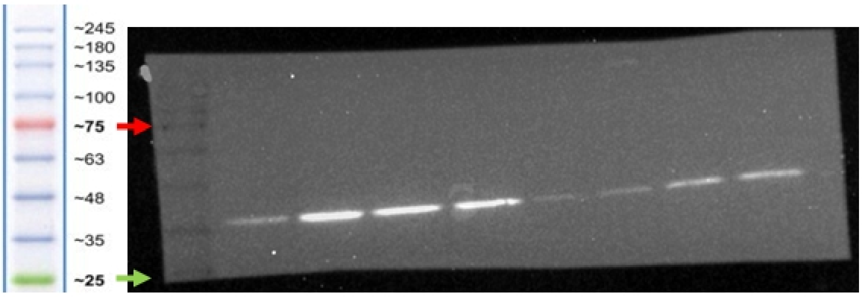

CHOP (line G361/A375)

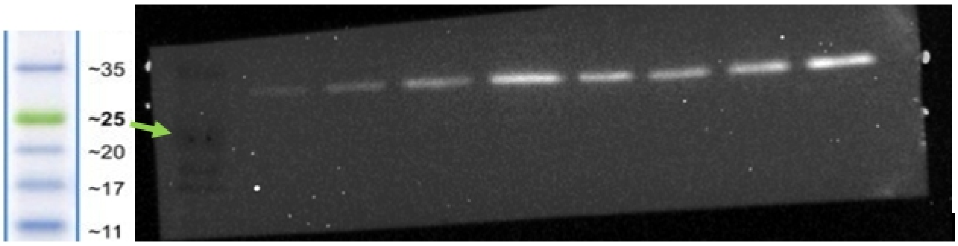

BiP (line G361/A375)

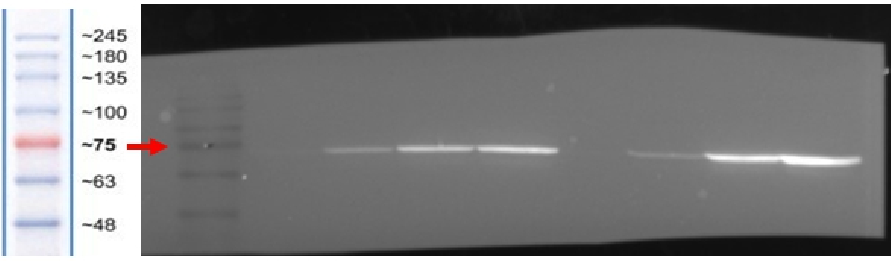

Figure S1. Actual photos of Western blot membranes to Figure 4

BCL-2 (line G361/A375)

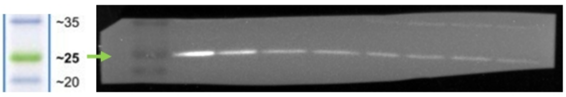

NOXA (line G361/A375)

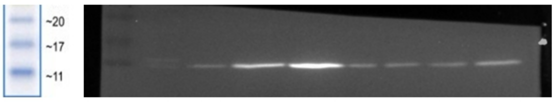

BIM (line G361/A375)

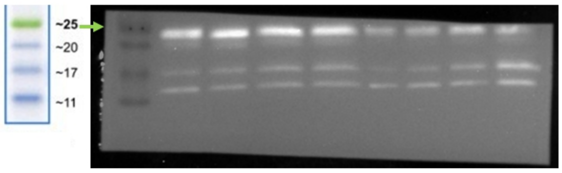

cl-Cas 8 (line G361/A375)

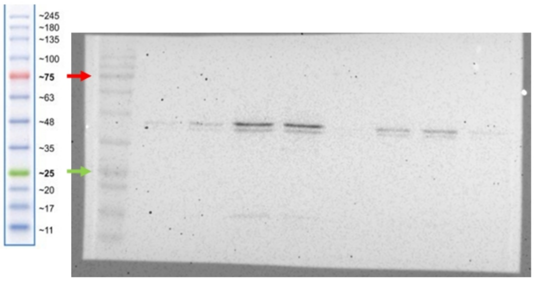

BID (line G361/A375)

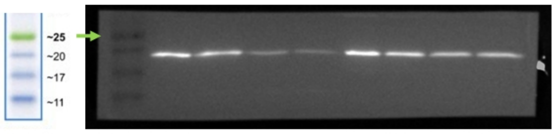

Cyt C (line G361/A375)

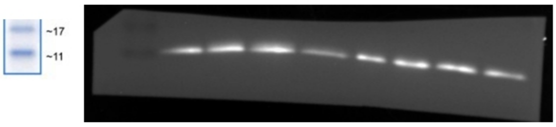

cl-Cas 9 (line G361/A375)

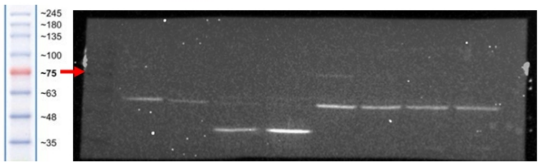

cl-Cas 3 (line G361/A375)

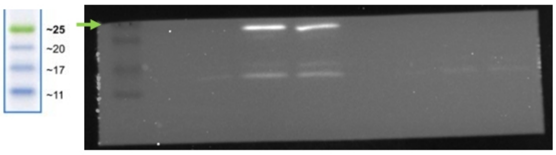

cl-PARP (line G361/A375)

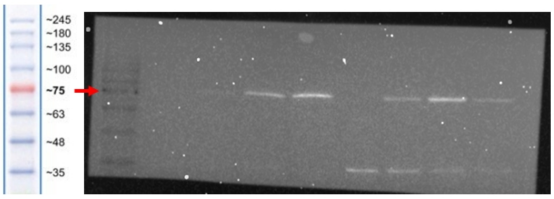

Figure S2. Actual photos of Western blot membranes to Figure 5
